# The use of wearable/portable digital sensors in Huntington's disease: A systematic review

**DOI:** 10.1016/j.parkreldis.2021.01.006

**Published:** 2021-02

**Authors:** Rosanna Tortelli, Filipe B. Rodrigues, Edward J. Wild

**Affiliations:** UCL Huntington's Disease Centre, UCL Queen Square Institute of Neurology, University College London, London, UK

**Keywords:** Huntington's disease, Biomarkers, Digital technology, Wearable sensors, Portable sensors

## Abstract

In chronic neurological conditions, wearable/portable devices have potential as innovative tools to detect subtle early disease manifestations and disease fluctuations for the purpose of clinical diagnosis, care and therapeutic development. Huntington's disease (HD) has a unique combination of motor and non-motor features which, combined with recent and anticipated therapeutic progress, gives great potential for such devices to prove useful. The present work aims to provide a comprehensive account of the use of wearable/portable devices in HD and of what they have contributed so far. We conducted a systematic review searching MEDLINE, Embase, and IEEE Xplore. Thirty references were identified. Our results revealed large variability in the types of sensors used, study design, and the measured outcomes. Digital technologies show considerable promise for therapeutic research and clinical management of HD. However, more studies with standardized devices and harmonized protocols are needed to optimize the potential applicability of wearable/portable devices in HD.

## Highlights

• Wearable/portable sensors have been proposed to detect and quantify manifestations of many neurodegenerative diseases.
• No systematic review so far has examined their use in Huntington's disease (HD).
• This work draws a broad picture of the digital wearable-based landscape in HD.
• The utility of wearables in clinical practice and therapeutic research still needs to be proved.
• Collaborative efforts are needed to further investigate their clinical use in HD.

## 1 Introduction

Huntington's disease (HD) is an autosomal dominant neurodegenerative disorder caused by an expanded trinucleotide CAG repeat in the *HTT* gene [1]. Clinically it is characterized by motor, behavioural, and cognitive signs and symptoms.

The natural history of *HTT* expansion carriers is divided into premanifest and manifest phases, with “clinical onset” diagnosed on the basis of “unequivocal” motor signs such as chorea [2,3]. However, a long prodromal phase, lasting a decade or more, frequently precedes this point and brings subtle motor, cognitive and behavioural features that can nonetheless be disabling [4].

Furthermore, signs and symptoms in HD can be extremely heterogeneous among patients and can also vary over time in the same patient in a non-linear manner. For example, motor impairment can range from the classical hyperkinetic involuntary movements to a more subtle hypokinetic impairment of voluntary movements, as well as impairment of motor coordination [5]. Additionally, signs and symptoms can also display short-term fluctuations.

Phenotype variability and the difficulty in consistently detecting subtle early clinical manifestations pose challenges to therapeutic development as well as clinical management. The Unified Huntington's Disease Rating Scale Total Motor Score (UHDRS TMS), has been “recommended” by the International Parkinson and Movement Disorder Society (MDS) for the assessment of motor signs in HD [6] and included by the National Institute for Neurological Disorder and Stroke HD group in a list of recommended sensitive outcome measures to be used as primary or secondary endpoints HD clinical trials [7]. However, its use in clinical trials has shown limited sensitivity, especially in the pre-manifest stage of HD [3,8]. It is also unreliable in capturing day-to-day or minute-to-minute variability of motor signs which could easily dwarf any treatment effect. In addition to more reliable measures, there is therefore face value in assessing manifestations of HD over a longer period, with high-frequency or continuous monitoring.

Quantitative measures of motor and cognitive alterations in HD can be an optimal tool to detect and monitoring subtle modifications even in pre-manifest HD [9,10]. However, such quantitative assessment is mainly based on expensive and cumbersome technology that can only be used in-clinic settings for limited time periods [11].

Recently, advances in wearable/portable sensors, information and communication technologies, have enabled a continuous monitoring of chronic diseases. The use of wearable/portable sensors allows the collection of high-dimensional data from multiple domains and during everyday activities, in order to obtain a detailed, objective and precise picture of disease manifestations. In addition, GPS data can provide evidence on real-world mobility and be a surrogate of social activity, while smartphones and other devices can also be used to implement questionnaires about symptoms or cognitive tasks. The high spatial and temporal resolution of the registered data allows the monitoring of long-term trends and short-term fluctuations of symptoms, as well as the detection of “soft” signs and symptoms of disease onset/progression, or of therapeutic response that would otherwise go unnoticed. By improving signal to noise ratios, this could be useful to increase the power of clinical trials for new drug discovery. The term ‘digital biomarkers’ is sometimes used to denote the meaningful outputs derived from electronic sensor data, whether or not the equipment used is wearable/portable.

Such technologies are still in their infancy when it comes to implementation in such settings.

Wearable/portable sensors, have been already used in numerous neurological disorders, such as Parkinson's disease (PD) and Alzheimer's disease (AD) and other dementias [12,13] and in 2017 an Alzheimer's Association Research Roundtable concluded with a strong recommendation to pharmaceutical companies to include digital tools as secondary endpoints in AD clinical trials in parallel with other already accepted and widely-used measures [14].

We undertook a systematic review to provide a comprehensive overview of the use of such devices in HD and provide an evidence basis to comment on possible future directions.

## 2 Materials and methods

### 2.1 Search strategy and selection criteria

An electronic database search was performed on April 29th^,^ 2019 on MEDLINE, Embase, and IEEE Xplore in order to identify articles related to the use of wearable/portable sensors in HD. In line with the PRISMA statement [15], an additional manual search was performed among the references of selected articles.

We developed detailed search strategies for each database searched. Please see Appendix 1 for the MEDLINE search strategy, Appendix 2 for the Embase search strategy, and Appendix 3 for the IEEE Xplore search strategy. Duplicates were excluded automatically with EndNote X9 and manually during the study selection process, which was conducted on Rayyan QCR web application [16].

We included original articles and abstracts/conference proceedings of any language reporting studies performed in humans that investigated the use of wearable/portable sensors to assess motor, behavioural or cognitive signs/symptoms in pre-manifest and/or manifest HD. We excluded review articles or book chapters. A wearable device was defined as an electronic technology or computer designed to be worn on the body, or embedded into watches, bracelets, clothing, and similar items [17]. A portable device was defined as any device that can easily be carried or worn on a belt or in a pocket. Studies reporting quantitative motor or cognitive assessment in HD using non-wearable sensors (e.g.: GAITRite instrumented carpet [18] or the Saccadometer Advanced [19]) were excluded from this review.

### 2.2 Review process

Two review authors independently screened for eligibility the titles and abstracts of all identified references. The full-text of all potentially eligible reports were retrieved and screened using the same procedure. Disagreements were resolved by discussion, or by consulting a third author.

### 2.3 Validity analysis

We conducted a validity analysis of the included wearable/portable devices/tools. We followed the strategy proposed by the Movement Disorder Society Committee on Rating Scales Development to appraise clinical assessment tools in HD [6,[20], [21], [22], [23]]. We included seven criteria with a Yes/No/Not Applicable response, namely: 1- used in HD, 2- used in HD by more than one group, 3- test-retest reliability, 4- ability to discriminate cases from controls, 5- ability to capture disease stage/severity, 6- ability to capture changes over time, 7- ability to detect therapeutic response. The answer “Not Applicable” referred to the fact that criterion has never been investigated for that specific device/tool in HD.

## 3 Results

### 3.1 Search results

**Fig. 1:**
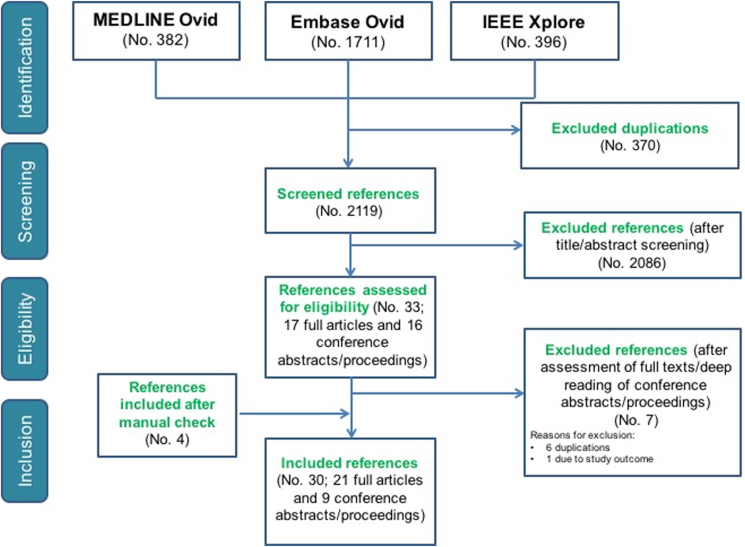
Flow-diagram for selection process.

The electronic search returned 2489 records (MEDLINE 382; Embase 1711; IEEE Xplore 396), resulting in 2119 records after removal of duplicates. Title and abstract screening excluded 2086 records not meeting the inclusion criteria. We assessed 33 full-texts, of which 16 were conference abstracts/proceedings and 17 were full-text original articles. Six conference abstracts were excluded because of duplications; and 1 conference abstract due to study outcome (presented no results). Additional four original articles were included after a manual search across the references of the assessed full-texts. At the end of the evaluation process, according to the eligibility criteria, 30 references were included in the final review (Fig. 1). Two references [24,25] refer to the same study, but present different analyses and results, so we did not consider them as duplicates.

### 3.2 General characteristics of the included studies

The main characteristics of the included studies are listed in Table 1. Twenty-one of them were published in indexed journals, while 9 were presented at international conferences [[26], [27], [28], [29], [30], [31], [32], [33], [34]]. The included studies cover an extensive time period, with three studies reporting the use of accelerometers before the year of 2000 [5,35,36].

**Table 1:** Summary of the included studies.

First author + YOP	Journal	n. of patients	n. of controls	Longitudinal	Type of sensor	Wearing position	Duration of use	Location of monitoring	Measured disease characteristics	Main results
Myers 1979 [36]	Biol Psychiatry	10 mHD, 15 at risk HD	0	no	Accelerometer	Not specified	Not specified	Clinic	Tremor	Accelerometer measures can detect and characterize tremor in manifest and pre-manifest HD
Folstein 1983 [35]	Neurobehav Toxicology and teratology	17 mHD, 27 at risk HD	10	no	Three-axial piezoelectric accelerometer (Wilcoxon Model no.139)	Dorsal surface of subject's hands	4 tasks, 5 10-s trials for each task	Clinic	Involuntary movements; some voluntary movements (simple reaction time, finger tapping, movement time)	Motor abnormalities can be detected in manifest and at risk HD; screening of motor abnormalities in the population
van Vugt 1996 [5]	Movement Disorders	14 mHD	14	no	Wrist-worn activity monitor (accelerometer) (Gaehwiler Electronic, Switzerland)	Non-dominant wrist	5 successive days and nights	Home	General daytime motor activity	Higher hypokinesia in HD patients
van Vugt 2001 [46]	Movement Disorders	64 mHD	67	yes	Wrist-worn activity monitor (accelerometer) (Gaehwiler Electronic, Switzerland)	Non-dominant wrist	5 successive days and nights	Home	General daytime motor activity	Higher hypokinesia in HD patients; correlation with impaired voluntary movements, disturbed posture and gait, and reduced functional capacity; progresses with functional disability
Hurelbrink 2005 [45]	J Neurol	8 mHD	8	no	Actiwatch-Neurologica (Cambridge Neurotechnology)	Preferred wrist	48 h	Home	Day- and night-time involuntary movements; Sleep-wake activity	Greater activity levels in HD while awake and during sleep; HD sleep longer than controls
Grimbergen 2008 [49]	Movement Disorders	45 mHD	27	no	Digitally-based angular velocity transducer (SwayStar)	Lower back	Time to walk on the GaitRite carpet)	Clinic	Trunk movements	Trunk displacement significantly greater in patients than controls; increased trunk sway in fallers compared to non-fallers; clinical chorea scores positive correlated to the range of angular trunk motion
Khalil 2010 [29]	JNNP 2010-EHDN suppl	10 mHD 5 pHD	6	no	AD_BRC sensor with a three-axial accelerometer	Sternum	Time of TUG performance	Clinic	Performance of Timed Up and Go Test	Accelerometer objective measures can be useful to catch disease specific features and so to differentiate between groups
Dalton 2013 [38]	Gait and Posture	14 mHD 10 pHD	10	no	AD_BRC sensor with a three-axial accelerometer	Chest	Unspecified (duration of the examination in clinic)	Clinic	Balance; gait	An accelerometer based sensor may be an effective means of differentiating between premanifest and manifest Huntington's disease subjects
Rudzinska 2013 [43]	Neurologia I Neurochirurgia Polska	43 DA 28 mHD 23 tic disorders	51	no	Three-axial accelerometer (BIOPAC)	Proximal phalanx of the third finger	1.5 min (accelerometer registration)	Clinic	Tremor	Postural and essential type tremor found in 10% of HD; prevalence of tremor is considerably higher among patients with degenerative ataxias compared with HD, tic disorder and the control group. The most common type of tremor accompanying ataxias, HD and tic disorders is essential tremor type
Norberg 2013 [26]	AFMR 2013 CA	15 PD or mHD	0	no	Wireless three-axial accelerometers (UCLAWireless Health Institute)	Both ankles	4 50-foot timed training walks + 3 days of monitoring	Clinic and home	Gait	Wireless sensors can obtain multiple measures of gait and other physical activities in an inexpensive and unobtrusive manner
Trojaniello 2014 [33]	IEEE Conference 2014	10 mHD	10	no	MIMU (Opal, APDM, Inc)	Ankle	1 min walking	Clinic	Gait	The MIMU has about 30% of errors associated to the best estimates of gait direction changes for patients, compared to gold standard (GAITRite Math)
Collett 2014 [37]	Gait & Posture	7 pHD 28 mHD	22	no	IMU (Pi-node Philips, Netherlands)	Taped over the fourth lumbar vertebra	8.8 or 10 m walking	Clinic	Gait	More variability in gait parameters in mHD compared to controls; no differences between pHD and HC, except for 1 parameter of the phase plot analysis, which also correlated with UHDRS-TMS and DBS. Phase plot analysis as a sensitive method to detect gait changes in HD
Trojaniello 2015 [42]	Gait & Posture	10 stroke 10 PD 10 mHD	10	no	MIMU (Opal, APDM, Inc)	Over the subject lumbar spine, between L4 and S2	1 min walking	Clinic	Gait	Comparison of 3 different methods to detect gait events. None of the tested methods outperformed the others in terms of gait parameter determination accuracy. Missed or extra gait events were found for all methods where pathological populations were analysed
Hogarth 2015 [28]	ICPDMD 2015	5 mHD	5	no	Shoe-worn inertial sensor (APDM Inc)	Both shoes	walking hours for 7 days	Home	Gait	Gait parameters correctly identified subjects. Significant differences between HD and HC in gait parameters
Townhill 2016 [40]	J Neurosci Meth	9 mHD 4 pHD	9	no	Actiwatch-Neurologica (Cambridge Neurotechnology) + ambulatory EEG	Non-dominant wrist	24 h (EEG); 7 days continuously (Actiwatch)	Home	Circadian Rhythm	Actiwatch is not a reliable tool for measuring awake/sleep periods in patients with movement disorders; no differences in circadian rhythmicity between groups
Andrzejewski 2016 [48]	J of HD	15 mHD	4	no	Accelerometer-based wearable PAMSys-X (BioSensics, Cambridge, MA)	Both ankles, both wrists, and chest	7 days	Clinic and home	General daily motor activity; gait	Same level of physical activity; differences in gait measures between HD and controls; feasible use of wearable sensors
Mannini 2016 [44]	Sensors	17 mHD 15 post-stroke	10	no	MIMU (Opal, APDM, Inc)	Both ankles, and over the subject's lumbar spine between L4 and S2	Unspecified (duration of the examination in clinic)	Clinic	Gait	Propose and validation of a new machine learning framework for gait classification (normal vs pathological)
Dinesh 2016 [25]	IEEE Xplore Digital Library	16 PD 10 mHD	15	no	Accelerometer-based BioStampRC wearable sensors, MC10 Inc (Lexington, MA)	Both anterior thighs, both proximal anterior forearms, and medial chest	2 days	Clinic and home	Gait	Signal analysis of light-weight body-affixed sensors can detect motor symptoms associated with PD and HD
Bennassar 2016 [27]	Procedia Computer Science (20th International Conference on Knowledge Based and Intelligent Information andEngineering Systems)	15 mHD	7	no	GENEActiv three-axial accelerometer (Activinsights Ltd, Cambridgeshire, UK)	Both wrists, and chest	Few minutes (time of completing the Moneybox-Test tasks)	Clinic	Movements of the upper limbs during the execution of the Money Box Test	Introduction of a new approach to automatically classify HD and controls (upper-limb movements)
Kegelmeyer 2017 [50]	J Neurol Sci	41 mHD	36	no	iPod with the Level Belt Pro software installed	Back at the level of L5 and of the lower border of scapulae	Unspecified (duration of the examination in clinic)	Clinic	Trunk control	Significant greater amplitude of thoracic and pelvic movements in HD vs controls (++ in static than in dynamic tasks)
Maskevich 2017 [39]	J of HD	4 pHD 3 mHD	0	no	Actiwatch Spectrum Pro (Philips/Respironics), Fitbit One and Jawbone UP2	Non-dominant wrist	Overnight	Clinic	Sleep characteristics	Three monitors less accurate of polysonnography to estimate sleep parameters in HD. Can't be a good replacement, but sufficient for overall estimations of sleep-wake patterns, and/or to assess gross level changes over time
Adams 2017 [24]	Digit Biomark	15 mHD 5 pHD 16 PD	20	no	Accelerometer-based BioStampRC wearable sensors, MC10 Inc (Lexington, MA)	Both anterior thighs, both proximal anterior forearms, and medial chest	2 days	Clinic and home	General daytime motor activity	Patients with HD spent more time lying down; participants happy with the sensors
Saadeh 2017 [32]	IEEE Conferences 2017	13 ALS, 20 mHD, 15 PD	16	no	Flexi-force sensing resistor (A201 Tekscan)	Shoe sole	Unspecified (used of an existing database?)	Clinic	Gait	The system classified the different groups with high sensitivity and specificity and a high classification accuracy
Youdan 2018 [30]	HSG 2018	37 mHD	15	no	MIMU (Opal, APDM, Inc)	Medial chest, medial lower back, both ankles and both wrists	Time of task performing	Clinic	Gait; cognition	Dual-task impairment in HD compared to HC, as showed by increased total sway area, decreased gait speed and decreased correct response to cognitive tasks
Waddel 2018 [34]	HSG 2018	14 subjects	?	yes	Android smartphone app (GEORGE)	Smartphone	1 month	Clinic and home	Gait, involuntary movements, voice, balance, dexterity, mobility, socialization	Feasibility of the app
Lipsmeier 2018 [31]	JNNP 2018-EHDN suppl	46 mHD	0	yes	Smartphone and Smartwatch (ROCHE platform)	Preferred wrist (smartwatch) and belt or trouser pocket (smartphone)	8- week preliminary results	Home	General daytime motor activity; motor tasks; chorea; balance; cognition; mood; quality of life	Good adherence; feasibility
Lauraitis 2018 [47]	IEEE j of Biomedical and Health Informatics	11 mHD	11	no	Android tablet app	Tablet	Once or twice a week for an unspecified period	Home	Motor and cognitive abilities trough three tasks	High classification accuracy of the app and useful support for automated medical examination
Acosta-Escalante 2018 [52]	IEEE Special edition on trends, perspectives and prospects of machine learning applied to biomedical systems in internet of medical things	7 mHD	7	no	Movement sensors on two smartphones iPhone 5S	Both ankles	Walking on a 20-m math during visits of 7 consecutive days	Clinic	Gait	Meta-classifier algorithms useful for improving accuracy in classification and reducing the number of sensor devices needed. Best performance of Logitboost & RandomForest combination
Bennasar 2018 [51]	IEEE transactions on neural systems and rehabilitation engineering	44 mHD	48	no	Three-axial accelerometer GENEactiv	Both wrists, and chest	Few minutes (time of completing the Moneybox-Test tasks)	Clinic	Movements of the upper limbs during the execution of the Money Box Test	Presentation of a system for an objective and continuous assessment of motor impairment during a novel upper limb task for HD patients
Bartlett 2019 [41]	Neurobiol of Sleep and Circadian Rhythms	32 pHD	29	no	Wrist-worn actigraphy GT3X + ActiGraph monitor	Non-dominant wrist	7 nights	Home	Circadian rhythm and habitual sleep characteristics	Decreased habitual sleep efficiency and increased awakenings in pHD compared with HC. No association between hypothalamic volume and circadian rhythm or habitual sleep outcomes in pre-HD

**YOP**: year of publication; **HD**: Huntington's disease; **mHD**: manifest Huntington's disease; **pHD**: pre-manifest Huntington's disease; **PD**: Parkinson's disease; **DA**: degenerative ataxia; **ALS**: amyotrophic lateral sclerosis; **HC**: healthy controls; **IMU**: inertial measurement unit; **MIMU**: magnetic inertial measurement unit; **UHDRS-TMS**: unified Huntington's disease rating scale – total motor score; **DBS**: disease burden score.

The majority of the studies were focused on manifest HD, with only six including pre-manifest HD participants [24,29,[37], [38], [39], [40]], one focusing on pre-manifest only [41], and two, performed before the availability of the HD genetic test, involving “at-risk” individuals [35,36]. Six studies also included patients with other neurological diseases, like PD [[24], [25], [26],^32^,^42^], degenerative ataxia [43], tic disorders [43], stroke [42,44], and amyotrophic lateral sclerosis [32]. All studies but four [26,31,36,39] compared the patient data with healthy volunteers. The study setting was “in-clinic” for 17 of the included studies, at the participant's home for 8 [5,28,31,40,41,[45], [46], [47]], and both in-clinic and at home for the remaining four [[24], [25], [26],^34^,^48^]. The monitoring duration ranged from a few minutes (in-clinic studies) to 8 weeks in the home environment [31]. All studies but three [31,34,46] were cross-sectional. The mean follow-up was 2.0 years in one [46], not specified in another [34], while Lipsmeier et al. although with a longitudinal study design, only reported preliminary results of 8 weeks of monitoring [31].

### 3.3 Types of sensors

**Fig. 2:**
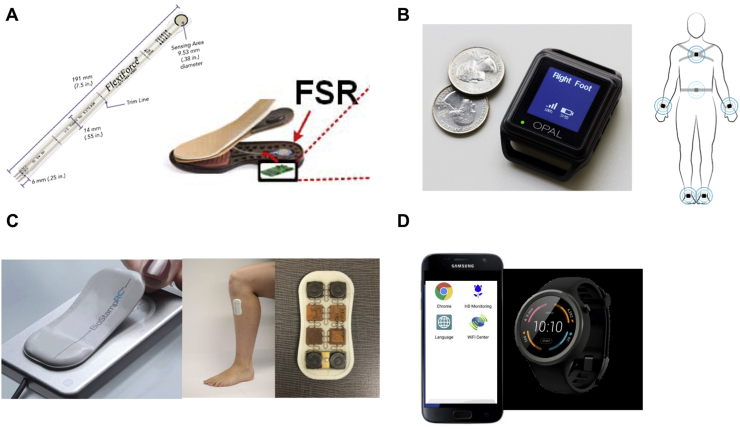
Examples of wearable/portable sensors used in Huntington's disease. **a.** Flexi-force sensing resistor (FRS), https://ww.tekscan.com/products-solutions/force-sensors/a201; **b.** Magnetic and inertial measurement unit (MIMU) (Opal™, APDM, Inc, Portland, OR, USA); **c.** Multi-mode adhesive flexible sensors (BioStampRc sensors, MC10 Inc, Lexington, MA, USA); **d.** Smartphone and smart-watch used for the Roche HD Digital Monitoring Platform^1^.

Accelerometers were the type of sensors used most, initially uniaxial and later mainly tri-axial. Only Saadeh and colleagues [32] proposed the use of a Flexi-force sensing resistor (FSR: https://ww.tekscan.com/products-solutions/force-sensors/a201. Fig. 2a), a thin, flexible piezoresistive force sensor. The sensor was placed unobtrusively into the shoe sole, and was able to translate the force applied in a designed sensing area into gait data, subsequently acquired and processed in a detection processor able to extract the discriminating features to classify different neurodegenerative diseases. The acquired information was then transferred to a mobile phone through a Bluetooth/Cloud network [32]. The studies of Waddel and colleagues and Lauraitis and colleagues didn't use any kind of motor sensor and they were based on an app for smartphone or tablet [34,47].

With advances in technology, the tested devices became lighter, smaller, and characterized by higher sample frequency, longer life and bigger memory capacity. Furthermore, they became flexible and dynamic. Other inertial measurement modules, such as gyroscopes, were added to accelerometers. This allowed the collection of data about rotation around three axes in addition to linear acceleration. Trojaniello and co-workers, Mannini et al. and Youdan et al. used a magnetic and inertial measurement unit (MIMU) (Opal™, APDM, Inc, Portland, OR, USA. Fig. 2b) attached to the subject ankles, wrists and lumbar spine and able to measure accelerations, angular velocities and local magnetic fields [30,33,42,44]. Dinesh and colleagues and Adams and colleagues used technologically advanced multi-mode adhesive flexible sensors (BioStampRc sensors, MC10 Inc, Lexington, MA, USA. Fig. 2c) with a weight of only 7 gr, and the possibility to operate in different modes including accelerometer, electrocardiogram, electromyography, and gyroscope functions [24,25]. They could be positioned on several parts of the body, like regular plasters, being unobtrusive and well-tolerated by the participants [24].

Successive iterations made devices easier to wear and more comfortable. The sensors used by Folstein and colleagues (dimensions: 3 × 3 × 6 cm) needed to be taped to the dorsal surface of both the subject's hands [35]; the Opal APDM sensors used by Trojaniello et al. Mannini et al., and Youdan et al. were smaller (dimensions: 4.8 × 3.6 × 1.3 cm) but still need to be strapped at the subject ankles, wrists or over the subject lumbar spine with a semi-elastic waist belt [30,33,42,44]. In the same way, many other proposed IMUs and sensors needed to be strapped at other body regions [29,37,38,43,48,49]. Kegelmeyer et al. used two iPods attached to two belts [50]. It is evident that all these solutions encompass a certain grade of discomfort for the participant and preclude the wide use of the sensors in the home environment, during the activities of daily living and for a long time interval. Later studies used adhesive sensors or wrist-worn watch-type devices that can be worn with the minimum discomfort. Hogarth and colleagues used devices fitted into shoes [28]. Lipsmeier and colleagues proposed the use of paired smart-watches and smartphones that can be easily worn in social situations (Fig. 2d) [31], as well as other studies used small wrist-worn actigraphy devices [[39], [40], [41]].

### 3.4 Measured disease characteristics

**Fig. 3:**
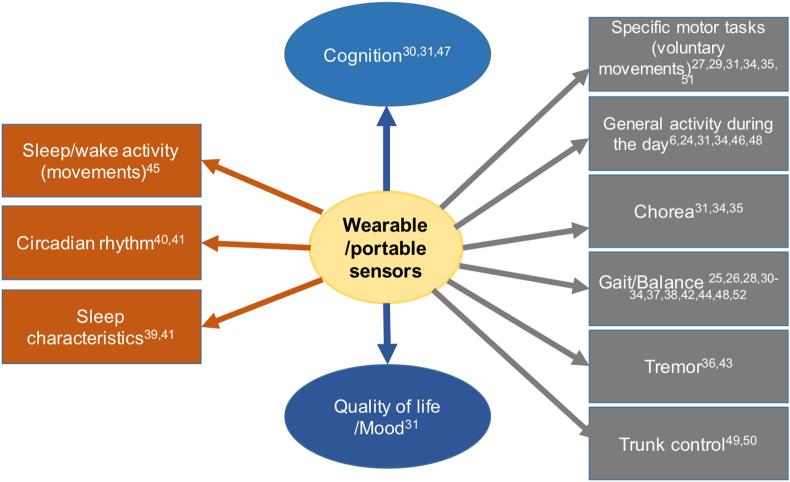
Disease characteristics investigated using wearable/portable sensors in HD.

All the investigated disease characteristics are graphically summarised in Fig. 3. The range of motor characteristics quantitatively measured by wearable tools in HD encompassed both involuntary and voluntary movements. Measured voluntary movements included specific tasks, such as finger tapping, reaction time and movement time [35], Timed up and go test [29,30], and Money Box Test as a measure of upper limb motor activity [27,51], or other structured motor tasks [24]. Other studies used wearable sensors to monitor sleep-wake activity (time spent asleep and motor activity during sleep) [45], as well as sleep measurements (total sleep time, sleep latency, sleep efficiency, and wake after sleep onset) [39], or circadian rhythm [40,41]. Several studies have investigated balance [38] and walking/gait characteristics [25,26,28,30,32,33,37,38,42,44,48,52]. Kegelmeyer et al. made a quantitative biomechanical assessment of trunk control, measuring the trunk stability during standing, sitting and walking, and the ability of individuals to modify trunk position responding to some auditory cues [50]. Other studies considered a more general concept of “activity level” during the performance of daily activities [5,46] and quantitatively assessed the daytime motor activity in a passive monitoring mode [24,48]. The study proposed by Lipsmeier et al. using a wearable smartwatch and a portable smartphone, was the first to provide a combination of passive monitoring and active tests, both in clinic and in the home setting [31]. The active tests, performed using a portable smartphone app, included questionnaires about mood, quality of life, and general wellbeing; cognitive tests, namely the Symbol Digit Modalities Test and the Stroop Word Reading Test; motor tasks, such as the Speed Tapping Test, the Draw a Shape Test, the Chorea Test, the Balance Test, and the U-Turn Test. Furthermore, the smartphone was equipped with a GPS, in order to register the daily activities of the participants (Fig. 3). Both the devices were designed for long-term monitoring and able to directly transmit the acquired data when connected to a Wi-Fi network [31]. Also the smartphone app proposed by Waddel et al. contained tests to assess several disease characteristics, like gait, chorea, voice, balance, dexterity, bodily motion, and socialization [34], whereas the tablet app proposed by Luraitis and co-workers was able to track tremor and cognitive impairment using three tasks with touch and visual stimulus modalities [47].

Finally, only one study investigated the participants' experience with the sensors through an electronic survey about comfort, security of adhesion, and removal of sensors [24]. They showed that the majority of participants found the sensors “comfortable” and “easy to remove”, while there was a general dissatisfaction about the sensors’ adhesion [24].

### 3.5 Performance of wearable devices in HD: what did they add to our knowledge?

Despite the increasing use of wearable/portable sensors in HD, their contribution in understanding the natural history of the disease or in better defining disease characteristics is still limited. Some of the studies have been focused on evaluating the sensor performance and the level of agreement between the registered parameters and some gold standards. Gait parameters measured by wearable/portable sensors have been demonstrated having a strong agreement with gold standard measurements, such as the GAITRite mat [38]. On the other hand, wearable devices used for the assessment of circadian rhythm or sleep-wake activity, produced poor agreement with the gold standard, polysomnography, especially in identifying the awake periods in both asymptomatic and symptomatic individuals. The study of Townhill and colleagues demonstrated that the Actiwatch Activity monitoring system (Cambridge Neurotechnology Ltd) overestimated periods of wakefulness compared to EEG data [40]. Maskevich and co-workers showed that both commercially available activity monitors (Fitbit and Jawbone) and a research-based actigraph (Actiwatch Spectrum Pro, Philips/Respironics, Murrysville, PA), presented low-agreement with polysomnography, significantly overestimating or underestimating different sleep parameters [39]. Nevertheless, they have been used in a few studies, demonstrating the general utility of actigraphy in distinguishing between manifest HD and controls, with HD patients sleeping for a longer time period compared to controls and presenting a higher percentage of involuntary movements during sleep [45], and even between pre-HD and controls based on sleep efficiency [41].

The most interesting, common and reproducible information that wearable/portable technologies have added to the HD field so far is related to their utility in automatically distinguishing between patients and controls based on features of a specific trait or disease characteristic. The most investigated trait has been gait/walking ability. Spatio-temporal gait parameters, like velocity, step length, stride length, gait symmetry/regularity and postural sway, derived by tri-axial accelerometers or inertial sensors, were able to differentiate manifest HD from pre-manifest HD and/or healthy controls [28,38]. The discrimination ability of gait parameters between HD patients and healthy controls seems to increase at home with a longer period of observation. Andrzejewski and colleagues showed that during the in clinic visit, step time variability was increased in HD, compared to controls, while at home differences were observed for all the considered gait parameters [48]. In addition, in the home setting, all the analysed gait measures were able to differentiate HD based on their level of motor impairment (i.e. patients with TMS <50 from those with TMS ≥50) [48]. So, in the home setting, the variability of the motor measures detected by the sensors was generally greater that those observed in the controlled clinical environment, and with more observations at home, additional differences in gait were detected [48]. Collett et al. proposed the measurement of gait variability parameters as a tool to discriminate between manifest HD, pre-manifest HD and controls, showing that manifest HD patients presented a higher gait variability compared to pre-manifest and healthy controls [37]. Interesting, one of the parameters of gait variation (Ratio ∀, namely the ratio between the spatiotemporal variability and the temporal variability of consecutive wave forms from vertical movements of a walk test) was also smaller in pre-HD compared with controls and showed a high discrimination ability between the two groups (AUC = 0.81) [37].

Other movement features extracted from wearable/portable sensors have been investigated and proposed as potentially able to automatically and accurately classify HD and controls. Among those, selected features extracted from the accelerometer data registered during a multitasking active test for upper limbs (namely the Money Box Test) [27,51], specific trunk movements [50], and angular trunk displacement [49] were the most interesting. Grimberger and co-workers showed that patients with HD had greater angular trunk displacement compared with controls and this increase in trunk sway was more pronounced in fallers than in non-fallers and positively correlated with clinical chorea scores [49]. In the study of Kegelmeyer and colleagues, wearable accelerometers were used for rehabilitation purposes in order to adjust trunk movements and reflexes in HD patients [50]. Youdan and colleagues showed dual-task impairment in HD, reporting an increased total sway area, decreased gait speed and decreased correct response to cognitive tasks in HD participants who performed motor and cognitive tasks at the same time [30].

Extracting meaningful and useful outcomes from high-dimension datasets is a major challenge as digital biomarker technology becomes ever more complex. That was the reason why some of the studies focused on advanced machine learning approaches and new algorithms or analysis methods to extract parameters with the best discrimination ability and increase the classification accuracy between HD and controls [25,32,37,44,51,52]. However, none of the proposed algorithms has been reproduced in a replication cohort.

### 3.6 Validity analysis

The results are reported in Supplementary Table 1. Only one of the included devices/tools fulfilled more than 3 of the proposed criteria [5,46]. The majority of them had a positive response to two criteria over seven. Six of them were positive to three criteria, and five of them to one only.

## 4 Discussion and future directions

This work provides a comprehensive overview of the wearable/portable sensors applied for the measurement of several disease characteristics in HD patients, both in the pre-manifest and manifest stages of the disease.

This topic has risen in prominence during the COVID-19 pandemic, in which digital and remote healthcare and monitoring technologies have been increasingly leveraged in order to provide care and clinical trial continuity while minimising viral transmission; it is probable that such technologies will continue to be used to a higher extent than before the pandemic [53].

Our results confirm that, in common with other neurodegenerative diseases, wearable/portable technologies are of large interest in HD, so far mainly as a tool for automatic discrimination of patients from healthy subjects, and to detect early signs and symptoms of the disease. It is now clear that measurements of involuntary movements as well as of other disease characteristics like trunk sway or sleep patterns/movements using wearable/portable devices can be a reliable approach to identify patients in the manifest stage of the disease and they are promising in the characterization of the pre-manifest and early manifest phases as well. This is of a huge interest because advanced wearable technologies represent a revolutionary approach in collecting data. They are able to measure objective parameters in a tolerable way and to collect a large amount of data in “ecological” environments, like homes or community settings in order to reduce measurement errors of in-clinic assessments [54,55]. Furthermore, wearable sensors and systems are able to maximize the temporal and spatial resolution of motor and non-motor phenomena that are expected to change over time, to be rare and occasional, or to happen by definition over long time periods [56], providing a more accurate and realistic report of the behaviour of interest [57].

However, in the current scenario, as highlighted by the results of the validity analysis, a major pitfall for the applicability of wearables/portables in clinical practice and therapeutic investigations is the lack of validation of the proposed devices. The majority of them have been used in a single population, with no data about reliability and reproducibility of the acquired data and derived results [58]. Most studies used different hardware and methods, so the wearable devices and acquired data cannot be readily compared, and most of the studies lacked a validation cohort. Another limitation is the fact that the methodologies for the analysis of the huge amount of collected data to obtain meaningful disease-related signal from background noise, are a completely open field of discussion as well [56]. Furthermore, as with any rapidly growing field of interest, there is no gold standard for the validation of new proposed monitoring systems. Quantitative motor systems, such as GAITRite mats, can be a good gold standard for wearable sensors which measure gait parameters, but there are no corresponding reference electronic quantitative measures for wearables which measure other disease characteristics. On the other hand, the use of clinical scales as gold standards for validation of the proposed devices and collected features has several limitations related to the discrete and rater-dependent nature of these scales and to their low temporal and spatial resolution [45,59,60]. Finally, in the use of wearables/portables, selection bias must be considered. Socio-cultural factors such as age and enthusiasm for technology may influence recruitment and there is a lack of studies concerning the influence of relatives, gender, education, and working condition on the use of wearable/portable technologies. Furthermore, disease stage and functional status can play a role, as wearable/portable devices may not have the same applicability or tolerability across all disease stages.

All these limitations, as long as the lack of integration and standardization of the measured characteristics, are the major pitfalls responsible of the considerable distance between the very promising role of wearable/portable sensors and other digital technologies in neurodegenerative disorders, and their real adoption in clinical practice or in pharmacological studies [61]. Despite at least two decades of wide spread of wearables and huge advances in technologies, they have been only sporadically used as surrogates or exploratory end points [62,63].

### 4.1 Future directions

To advance the clinical applicability and utility of wearables/portables in HD there is an urgent and essential need for standardization, harmonization, openness and validation of the devices already available, which must be balanced with the pilot testing of successive generations of new devices. A major effort towards international collaborations and standardized and harmonized protocols for acquisition and analysis of data is needed, to avoid duplication of investments and unnecessary burden on patients, to integrate the best from different systems into a standard and easily accessible platform, and to increase the number of study participants and the validity of the results. PD sets a positive example here. A Task Force on Technology was created within the MDS in 2015 (https://www.movementdisorders.org/MDS/About/Committees--Other-Groups/MDS-Task-Forces/Task-Force-on-Technology.htm) with the main aim of maximizing the diagnostic and therapeutic potential of technology in the care of patients with movement disorders [56]. Furthermore, in 2019, the same task force proposed a roadmap to implement patient-centred digital outcome measures obtained using mobile technologies in PD [61]. They listed four “unmet needs” for mobile technologies: 1- Defining relevant patient-centred digital targets and outcomes to be captured with mobile health technologies (What to measure), 2- Selection criteria to guide the choice of mobile health technology (How to measure), 3- Web-based, open-source, modular, scalable and secure platforms for data analysis, integration, and visualization (What to display), 4- Establish a roadmap for regulatory approval and adoption into health care systems (How to disseminate). Subsequently they proposed a roadmap to satisfy those needs, but discussed that several challenges must be fought before the roadmap could be transferred to the real world [61].

Aspects of HD that are currently under-investigated, such as non-motor symptoms, have the potential to be studied using wearable technologies as well, adopting a more comprehensive and holistic approach with the aim to measure a broader spectrum of HD features.

**Fig. 4:**
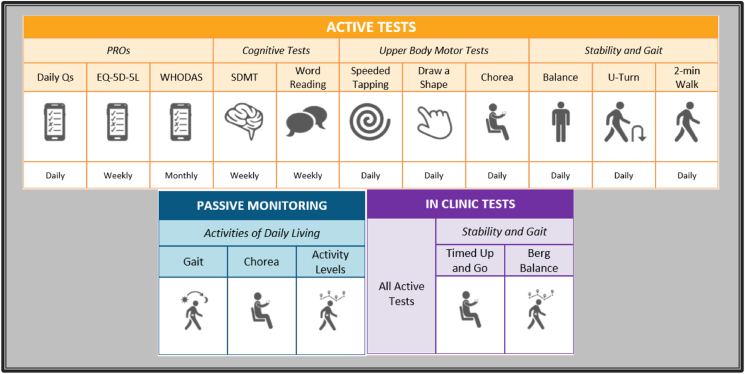
Graphic summary of all the tests^1^ (smartphone-based active tests, passive monitoring with wearables, and in-clinic tests) included in the Digital-HD study. **Daily Qs**: daily questions; **EQ-5D-5L**: Euro Quality of life - 5 Dimensions – 5 Levels questionnaire; **WHODAS**: World Health Organization Disability Assessment Schedule; **SDMT**: Symbol Digit Modalities Test.

There are two ongoing clinical, prospective, observational studies of advanced multimodal digital measurement systems. The first one is called “HD Wear - Wearable sensor system for monitoring Huntington's chorea during activities of daily living” and is a single-centre study conducted by the University of Rochester (NCT03599076). It started to recruit in mid-2018 and it is still recruiting at the time of writing. Its main aim is to develop a wearable sensor system for objective, sensitive, and continuous assessment of chorea in HD during activities of daily living. It is expected to enrol 50 participants (pre-manifest HD, manifest HD and healthy volunteers) and to monitoring them at home for 12 months. The second study is called “Digital-HD – Digital Biomarkers in Huntington's Disease”, a single-centre study, conducted at our institution – UCL Huntington's disease Centre – which aims to enrol 120 participants (40 manifest HD, 40 pre-manifest HD, and 40 healthy volunteers). The study design includes three in-clinic visits (baseline, 12 months and 18 months) and a continuous “passive monitoring” in the home environment wearing a smart-watch and carrying on a GPS-provided smartphone during routine daily activities. Furthermore, some daily smartphone-based “active tests” designed to measure a range of motor and non-motor symptoms in HD are also included (Fig. 4). The same platform is also part of two ongoing clinical trials in HD, namely GENERATION-HD1 (NCT03761849), a phase III multicentre randomized, placebo-controlled trial on the use of an antisense oligonucleotide against huntingtin mRNA, and GEN-EXTEND (NCT03842969), an open-label extension study regarding the same drug. This makes the platform the first to be tested in both observational and interventional settings in HD.

In summary, there is great promise that wearable and portable devices will contribute to a new digital era of biomarkers for HD, as well as in other neurodegenerative disorders. The availability of high-dimensional objective data with high spatial and temporal resolution is expected to increase the statistical power and interpretability of clinical trials and to reduce the sample size required to detect therapeutic effects [64]. They may eventually be used to guide collaborative decision making for patients and clinicians, but much work is required before such systems can be used as primary trial outcome measures or in the clinic.

## Funding

RT's salary is funded by a research grant from F. Hoffmann-La Roche to UCL.

EJW's salary has been funded by 10.13039/501100000265Medical Research Council, 10.13039/100005725CHDI Foundation, and F. Hoffmann La Roche.

## Authors’ contribution

EJW and RT conceived the study. FBR constructed and ran the electronic search. RT and FBR independently screened and selected the references. RT wrote the manuscript. EJW and FBR reviewed and revised the manuscript. All authors have approved the final article.

## Declarations of competing interest

RT, FBR and EJW are University College London employees.

EJW is the PI of the “Digital-HD study”, sponsored by 10.13039/501100000765University College London with a grant by Hoffmann-La Roche. RT and FBR are both involved in this study.

FBR has provided consultancy services to GLG and F. Hoffmann-La Roche Ltd.

EJW reports grants from, Triplet Therapeutics, 10.13039/100013223PTC Therapeutics, Shire Therapeutics, Wave Life Sciences, Mitoconix, Takeda, Loqus23. All honoraria for these consultancies were paid through the offices of UCL Consultants Ltd., a wholly owned subsidiary of University College London. 10.13039/501100008721University College London Hospitals NHS Foundation Trust has received funds as compensation for conducting clinical trials for 10.13039/100013669Ionis Pharmaceuticals, 10.13039/100004319Pfizer and Teva Pharmaceuticals.

## Appendix 1

MEDLINE search strategy.

1 exp Huntington Disease/
2 (Huntingto$ adj2 (disease or chorea)).ab,ti.
3 or/1-2
4 digital.tw.
5 exp Wearable Electronic Devices/
6 wearable$.tw.
7 sensor$.tw.
8 exp “Equipment and Supplies"/
9 device$.tw.
10 tracker$.tw.
11 accelerometer$.tw.
12 inertial measurement unit.tw.
13 smartphone$.tw.
14 gyroscope.tw.
15 or/4-14
16 and/3,15
17 (animals not humans).sh.
18 16 not 17

## Appendix 2

Embase search strategy.

1 exp Huntington Disease/
2 (Huntingto$ adj2 (disease or chorea)).ab,ti.
3 or/1-2
4 digital.tw.
5 exp Wearable Electronic Devices/
6 wearable$.tw.
7 sensor$.tw.
8 exp “Equipment and Supplies"/
9 device$.tw.
10 tracker$.tw.
11 accelerometer$.tw.
12 inertial measurement unit.tw.
13 smartphone$.tw.
14 gyroscope.tw.
15 or/4-14
16 and/3,15
17 (animals not humans).sh.
18 16 not 17

## Appendix 3

IEEE XPlore search strategy.

(huntington OR huntington's) AND (digital OR wearable OR sensor OR device OR tracker OR accelerometer OR gyroscope OR unit OR smartphone).

## Footnotes

1 Reproduced with permission from Hoffmann-La Roche
