# Supplementary material for: The use of wearable/portable digital sensors in Huntington's disease: A systematic review
Source: Parkinsonism Relat Disord. 2021 Feb;83:93–104. doi: 10.1016/j.parkreldis.2021.01.006 (PMC7957324; doi:10.1016/j.parkreldis.2021.01.006)
Supplement: Multimedia component 1 [file mmc1.docx]

**Supplementary Table 1**

| **Sensor/tool** | **Applied in HD** | **Applied by more than one group in HD** | **Test-retest Reliability** | **Ability to discriminate cases from controls** | **Ability to capture disease stage/ severity** | **Ability to capture changes over time** | **Ability to detect therapeutic response** |
| --- | --- | --- | --- | --- | --- | --- | --- |
| **Three-axial piezoelectric accelerometer (Wilcoxon Model no.139)[35]** | **Y** | N | N/A | **Y** | **Y** | N/A | N/A |
| **Wrist-worn activity monitor (accelerometer) (Gaehwiler Electronic, Switzerland)[5, 46]** | **Y** | N | N/A | **Y** | **Y** | **Y** | N/A |
| **Actiwatch-Neurologica (Cambridge Neurotechnology)[40, 45]** | **Y** | **Y** | N/A | **Y** | N/A | N/A | N/A |
| **Digitally-based angular velocity transducer (SwayStar)[49]** | **Y** | N | N/A | **Y** | **Y** | N/A | N/A |
| **AD_BRC sensor with a three-axial accelerometer[29, 38]** | **Y** | N | N/A | **Y** | **Y** | N/A | N/A |
| **Three-axial accelerometer (BIOPAC)[43]** | **Y** | N | N/A | N/A | N/A | N/A | N/A |
| **Wireless three-axial accelerometers (UCLAWireless Health Institute)[26]** | **Y** | N | N/A | N/A | N/A | N/A | N/A |
| **MIMU (Opal, APDM, Inc)[30, 33, 42, 44]** | **Y** | **Y** | N/A | **Y** | N/A | N/A | N/A |
| **IMU (Pi-node Philips, Netherlands)[37]** | **Y** | N | N/A | **Y** | **Y** | N/A | N/A |
| **Shoe-worn inertial sensor (APDM Inc)[28]** | **Y** | N | N/A | **Y** | N/A | N/A | N/A |
| **Accelerometer-based wearable PAMSys-X (BioSensics, Cambridge, MA)[48]** | **Y** | N | N/A | **Y** | N/A | N/A | N/A |
| **Accelerometer-based BioStampRC wearable sensors, MC10 Inc (Lexington, MA)[24, 25]** | **Y** | N | N/A | **Y** | N/A | N/A | N/A |
| **GENEActiv three-axial accelerometer (Activinsights Ltd, Cambridgeshire, UK)[27, 51]** | **Y** | N | N/A | **Y** | N/A | N/A | N/A |
| **iPod with the Level Belt Pro software installed[50]** | **Y** | N | N/A | **Y** | N/A | N/A | N/A |
| **Actiwatch Spectrum Pro (Philips/Respironics)[39]** | **Y** | N | N/A | N/A | N/A | N/A | N/A |
| **Flexi-force sensing resistor (A201 Tekscan)[32]** | **Y** | N | N/A | **Y** | N/A | N/A | N/A |
| **Android smartphone app (GEORGE)[34]** | **Y** | N | N/A | N/A | N/A | N/A | N/A |
| **Smartphone and Smartwatch (ROCHE platform)[63]** | **Y** | N | N/A | N/A | N/A | N/A | N/A |
| **Android tablet app[47]** | **Y** | N | N/A | **Y** | N/A | N/A | N/A |
| **Movement sensors on two smartphones iPhone 5S[52]** | **Y** | N | N/A | **Y** | N/A | N/A | N/A |
| **Wrist-worn actigraphy GT3X[41]** | **Y** | N | N/A | **Y** | N/A | N/A | N/A |
